# Cardiovascular disease risk and comparison of different strategies for blood pressure management in rural India

**DOI:** 10.1186/s12889-018-6142-x

**Published:** 2018-11-15

**Authors:** Devarsetty Praveen, David Peiris, Stephen MacMahon, Kishor Mogulluru, Arvind Raghu, Anthony Rodgers, Shailaja Chilappagari, Dorairaj Prabhakaran, Gari D. Clifford, Pallab K. Maulik, Emily Atkins, Rohina Joshi, Stephane Heritier, Stephen Jan, Anushka Patel

**Affiliations:** 1 The George Institute for Global Health, Hyderabad, India; 2 University of New South Wales, Sydney, Australia; 3 The George Institute for Global Health, University of New South Wales, Sydney, Australia; 4 The George Institute for Global Health, The University of New South Wales, Sydney, Australia; 5 The George Institute for Global Health, Hyderabad, India; 6 Institute of Biomedical Engineering, University of Oxford, Oxford, UK; 7 Centre for Chronic Disease Control, New Delhi, India; 8 London School of Hygiene and Tropical Medicine, London, UK; 9 Department of Biomedical Informatics, Emory University, Atlanta, GA USA; 10 Department of Biomedical Engineering, Georgia Institute of Technology, Atlanta, GA USA; 11 The George Institute for Global Health, Delhi, India; 12 Monash University, Melbourne, Australia

**Keywords:** Cardiovascular disease, Absolute risk, India, Treatment, Blood pressure

## Abstract

**Background:**

Non-optimal blood pressure (BP) levels are a major cause of disease burden globally. We describe current BP and treatment patterns in rural India and compare different approaches to BP lowering in this setting.

**Methods:**

All individuals aged ≥40 years from 54 villages in a South Indian district were invited and 62,194 individuals (84%) participated in a cross-sectional study. Individual 10-year absolute cardiovascular disease (CVD) risk was estimated using WHO/ISH charts. Using known effects of treatment, proportions of events that would be averted under different paradigms of BP lowering therapy were estimated.

**Results:**

After imputation of pre-treatment BP levels for participants on existing treatment, 76·9% (95% confidence interval, 75.7–78.0%), 5·3% (4.9–5.6%), and 17·8% (16.9–18.8%) of individuals had a 10-year CVD risk defined as low (< 20%), intermediate (20–29%), and high (≥30%, established CVD, or BP > 160/100 mmHg), respectively. Compared to the 19.6% (18.4–20.9%) of adults treated with current practice, a slightly higher or similar proportion would be treated using an intermediate (23·2% (22.0–24.3%)) or high (17·9% (16.9–18.8%) risk threshold for instituting BP lowering therapy and this would avert 87·2% (85.8–88.5%) and 62·7% (60.7–64.6%) more CVD events over ten years, respectively. These strategies were highly cost-effective relative to the current practice.

**Conclusion:**

In a rural Indian community, a substantial proportion of the population has elevated CVD risk. The more efficient and cost-effective clinical approach to BP lowering is to base treatment decisions on an estimate of an individual’s short-term absolute CVD risk rather than with BP based strategy.

**Clinical trial registration:**

Clinical Trials Registry of India CTRI/2013/06/003753, 14 June 2013.

**Electronic supplementary material:**

The online version of this article (10.1186/s12889-018-6142-x) contains supplementary material, which is available to authorized users.

## Background

Cardiovascular diseases (CVD) are a major cause of premature morbidity and mortality globally, with ischemic heart disease and stroke responsible for 24·4% of all deaths in 2010 [1, 2]. About 80% of these CVD deaths occur in low-and middle-income countries [3]. CVD rates have decreased in developed nations due to major public health interventions and better preventive treatment, but they are a major contributor to the increasing burden of non-communicable diseases in developing countries [4–6]. In India, CVD already cause about 27% of deaths, indicating a rapid pace of epidemiological transition [7].

Modifiable CVD risk factors (including tobacco use, physical activity levels, blood pressure [BP], blood cholesterol, and diabetes) are common and are amenable to effective public health and clinical interventions [8–11]. Traditionally, clinical management of CVD risk factors has been based on directing treatment at abnormal levels of individual risk factors (i.e. treating “hypertension” or elevated cholesterol levels), however guidelines worldwide are increasingly incorporating the estimation of absolute CVD risk to determine whether preventive drugs should be initiated [12–16]. Recently in India, the central government launched the National Program on prevention and control of Cancer, Diabetes, Cardiovascular diseases, and Stroke (NPCDCS). As India does not have locally developed algorithms to predict CVD risk, WHO/ISH risk charts for SEAR-D region (details are explained in the methodology section) are recommended for clinical use [17]. The program advocates the use of BP lowering drugs in patients with intermediate CVD risk *and* BP levels ≥140/90 mmHg and those with high CVD risk *and* BP levels ≥130/80 mmHg [17]. NPCDCS was initiated in 2010 and is being rolled out across India in a phased manner. Currently, the program has been implemented in 100 of a total 675 districts, but little is known about its adoption and the potential consequences.

The objectives of the current analyses were to describe the population distribution of BP and CVD risk in a large rural Indian community, to describe current BP treatment patterns, and to compare the efficiency of single risk factor and absolute CVD risk approaches to BP lowering in this setting.

## Methods

The data used in these analyses were collected as the baseline assessment of a cluster randomized trial (SMART*health* India) evaluating an intervention aimed at improving CVD risk management [18]. A full household survey was conducted in 54 villages in Andhra Pradesh, India between February 2014 and May 2014.

### Participant selection

A total of 18 Primary Healthcare Centers (PHCs), broadly representative of West Godavari District of Andhra Pradesh were selected for participation in the SMART*health* India trial. Of the four revenue divisions of West Godavari district, all except three PHCs belonging to the Narsapur revenue division were selected based on trial eligibility. A further three PHCs were included from the neighboring Eluru revenue division [18]. Three villages were randomly selected from each PHC and a census list comprising the age and sex of all residents was enumerated from the 54 villages. All eligible persons of age 40 years and above were identified and interviewed after obtaining written informed consent. If an individual was not available at the initial visit, up to three additional visits were made to obtain the required data.

### Data collection

A questionnaire was administered to collect information on socio-demographic variables, CVD risk factors, known chronic conditions and current drug treatments. Each participant had their BP recorded in the seated position using an automated sphygmomanometer (Model UA-767PBT-C40, A&D Medical, Tokyo, Japan) after at least 5 min of rest. The measurement was done thrice, with each measurement made at least 2 min apart. The average of the last two readings was considered for the study. Finger prick capillary blood glucose was estimated using a point-of-care device (Abbott FreeStyle Optium, Alameda, California, USA) with fasting status at the time of sampling recorded.

### Estimation of CVD risk

Ten year risk of fatal or non-fatal major CVD event (myocardial infarction or stroke) was estimated using algorithms based on the World Health Organization/ International Society for Hypertension (WHO/ISH) risk charts [19]. These are color-coded charts tailored to 14 WHO epidemiological sub-regions, including South-East Asian Region-D (SEAR D; includes India). The risk charts predict the risk of CVD based on age, sex, smoking status, level of blood cholesterol, BP, and absence or presence of diabetes. There are two types of charts available - high information charts, for use when blood cholesterol information is available, and low information charts in the absence of cholesterol information. As blood cholesterol was not measured in this study, the low information charts were used. These charts use categories of risk factors: age (40 to 49, 50–59, 60–69, 70 years, and older), sex (male, female), smoking (no, smoker, or ex-smoker < 12 months), systolic BP (< 129·9, 130 to 149·9, 150 to 169·9, and > 170 mmHg), and presence of diabetes (fasting blood glucose ≥126 mg%; non-fasting blood glucose ≥200 mg%) to estimate 10-year cardiovascular risk. The non-fasting blood glucose threshold used for diagnosis of diabetes is consistent with other population-based diabetes screening programs in India [20].

To estimate the population distribution of CVD risk, pre-treatment BP levels were imputed for all participants reporting use of BP lowering medications. This was done by using the method described by Wald and Law for calculating the pre-treatment systolic and diastolic BP levels, as 80% of the high-risk patients on BP lowering treatment in the current study were on a single drug regimen [21]. The risk charts allocate individuals into the 5 risks groups: < 10%, 10–19%, 20–29%, 30–39%, and ≥ 40%. Any patient with established coronary heart disease, cerebrovascular disease, peripheral vascular disease or with a BP > 160/100 mmHg are categorised as high risk without the need for formal risk assessment. The risk charts predict risk for individuals aged 40 to 79 years; for the current analyses, age was fixed at 79 years for individuals aged 79 or older.

### Estimation of treatment benefit with different strategies for BP lowering

First, we estimated the number of events that are likely to occur in the study population if it was fully untreated over the next ten years. In these analyses, pre-treatment BP levels were imputed for individuals taking BP lowering treatment. As the WHO/ISH charts assign an individual to a category of risk, rather than an integer, the median value for each risk category was used as an indicator of average risk in each category. For example, for the category of < 10% risk the mid-point value of 5% was used, for the category of 10–20% the mid-point value of 15% was used, and so forth. For the category of > 40% (which included those with known CVD or BP > 160/100), a mid-point value of 50% was used. The mid-point value of risk for each category was then applied to the number of individuals within that risk category, and combining these values provided a total number across the population.

The next step was to estimate the number of events that would be averted under different treatment paradigms. Numerous randomized trials have shown that BP lowering reduces the relative risk of CVD events across a broad range of initial BP or CVD risk levels [22–25]. We used a relative risk reduction of 15% based on recent analyses of the Blood Pressure Lowering Treatment Trialists’ Collaboration [25]. For examining risk-based treatment paradigms, we only used three categories of risk: participants with a 10-year risk of ≥30% were grouped along with those with established CVD and BP > 160/100 mmHg as “high risk”; participants with absolute risk of 20–30% as “intermediate risk”, and < 20% as “low risk”. Participants were also classified as having or not having “hypertension” based on BP thresholds of 140/90 mmHg. The BP lowering treatment paradigms that were compared to an untreated population were: 1) current practice; 2) treating people with “hypertension” using the 140/90 mmHg threshold; 3) treatment according to the new Indian NPCDCS guidelines (drug therapy recommended in patients with CVD risk 20–30% *and* BP levels ≥140/90 mmHg or CVD risk of ≥30% *and* BP levels ≥130/80 mmHg [17]; 4) treating everyone in the intermediate and high risk categories (regardless of BP level); and 5) treating only those in the high risk category (regardless of BP level).

### Indicative cost-effectiveness analyses

The cost-effectiveness of each of the strategies relative to current practice in terms of cost per disability adjusted life years (DALYS) averted were estimated. Costs included costs of CV medication and potential savings associated with reduced hospitalisation from CVD events over 10 years. Medication cost was assumed to be 10 cents per day based on the costs of antihypertensive medications in India [26] (for a total of 10 years in case of high risk) and cost of hospitalization was estimated to be 200 USD for each event [27]. DALYs were determined by dividing total burden in terms of disability adjusted life year lost due to coronary heart diseases with the total number of acute coronary events per year in India based on current burden of disease estimates [28, 29]. These were then applied to the estimates of CVD events averted with each strategy.

### Sensitivity analyses

Sensitivity analyses were performed with the following differing assumptions to those made for the primary analyses: 1) relative risk reduction of CVD events with BP lowering drug therapy (10% and 20%); 2) estimated pre-treatment BP levels for those already on treatment (10 mmHg lower SBP and 5 mmHg lower DBP; and 5 mmHg lower SBP and 3 mmHg lower DBP); and 3) average 10-year CVD risk for individuals with an estimated risk in the > 40% band using the WHO/ISH charts (40% and 60%).

### Statistical analysis

Post-stratification weights were used to adjust for the survey design and sampling methods, both at the PHC and the village level. The PHC weight was based on the ratio of the 24 eligible PHCs in the West Godavari region to the sampled 18 PHCs. Village weight was the ratio of the total number of villages in each PHC to the sampled three villages per PHC. The final weight was calculated as a product of the PHC weight and the village weight. This provided appropriate standardized estimates for the population of 24 PHCs of West Godavari region and minimized any potential sampling bias. The population distribution based on the age and sex of the weighted sample population was similar to the population distribution of the West Godavari district derived from the Government of India census data [30].

Statistical analyses were carried out using statistical package SAS 9·3 (SAS, NC, USA). The “Surveyfreq”, “Surveymean”, and “Surveyreg” procedures were used to include survey weights, where relevant. Overall and age and sex stratum- specific estimates of risk factor levels were reported. Means and proportions were presented with 95% confidence intervals. Risk factor levels were compared between groups using independent t-tests for continuous variables and chi-square tests for proportions. A *p*-value of < 0·05 was considered to indicate a result unlikely to have been observed by chance.

## Results

### Participant characteristics

The eligible study population (age ≥ 40 years) from 54 villages was 74,402. Data were collected from 62,254 (83·7%) participants; 10,332 (13·9%) were not available for interview and 1816 (2·4%) refused participation. Non-participants were on average younger (43·0 vs. 54·1 years, *p* < 0·001) and more likely to be male (51·6% vs. 46·8%, p < 0·001) than participants. Diabetes status was unknown in 60 individuals, thus data from 62,194 individuals (Table 1) were included in these analyses.

**Table 1:** – Characteristics of the study population^a^

		Overall (*n* = 62,194)	Male (*n* = 29,097)	Female (*n* = 33,097)	*p*-values
Age (years), mean (95% CI)		54·0 (53·7–54·3)	54·7 (54·4–55·1)	53·3 (53·0–53·6)	< 0·001
Female, % (95% CI)		53·2 (52·5–53·9)			
Currently smoking, % (95% CI)		21·9 (20·6–23·2)	41·0 (38·5–43·4)	5·1 (4·4–5·8)	< 0·001
Currently chewing tobacco, % (95% CI)		1·5 (1·2–1·8)	3·1 (2·4–3·8)	0·1 (0·0–0·1)	< 0·001
Established CVD, % (95% CI)		4·0 (3·7–4·3)	5·0 (4·6–5·3)	3·2 (2·8–3·6)	< 0·001
Myocardial infarction/angina, % (95% CI)		2·3 (2·0–2·6)	2·7 (2·4–3·0)	2·0 (1·7–2·3)	< 0·001
Stroke, % (95% CI)		1·8 (1·6–1·9)	2·3 (2·1–2·6)	1·2 (1·0–1·5)	< 0·001
Peripheral vascular diseases, % (95% CI)		0·1 (0·1–0·2)	0·2 (0·1–0·3)	0·1 (0·0–0·1)	< 0·001
Self-reported diabetes, % (95% CI)		11·6 (10·6–12·6)	11·3 (10·2–12·3)	11·8 (10·8–12·9)	0·143
All diabetes, % (95% CI)		18·0 (16·8–19·2)	17·8 (16·6–18·9)	18·3 (16·9–19·6)	0·250
SBP (mmHg), mean (95% CI)		126·3 (125·2–127·3)	124·2 (123·4–125·1)	128·1 (126·8–129·4)	< 0·001
DBP (mmHg), mean (95% CI)		79·8 (79·2–80·4)	79·4 (78·8–79·9)	80·3 (79·6–80·9)	< 0·001
BP lowering treatment, % (95% CI)		19·6 (18·4–20·9)	16·1 (15·1–17·2)	22·7 (21·2–24·2)	< 0·001
10-year adjusted cardiovascular risk, % (95% CI)^b^					
I	< 10% risk	63·9 (62·5–65·2)	63·3 (61·8–64·8)	64·4 (62·6–66·1)	0·168
II	10–20% risk	13·0 (12·5–13·5)	13·9 (13·0–14·7)	12·2 (11·7–12·6)	< 0·001
III	20–30% risk	5·3 (4·9–5·6)	5·9 (5·4–6·4)	4·8 (4·5–5·0)	< 0·001
IV	30–40% risk	1·2 (1·1–1·3)	1·8 (1·6–2·1)	0·6 (0·5–0·7)	< 0·001
V	> 40% risk	0·8 (0·8–0·9)	0·6 (0·5–0·7)	1·0 (0·9–1·2)	< 0·001
VI	Established CVD	4·0 (3·7–4·4)	5·0 (4·6–5·3)	3·2 (2·8–3·6)	< 0·001
VII	BP ≥160/100 mmHg	11·8 (11·1–12·5)	9·5 (8·9–10·1)	13·8 (12·9–14·8)	< 0·001
10-year adjusted cardiovascular risk groups, % (95% CI)^b^					
Low risk (I + II)		76·8 (75·7–78·0)	77·2 (76·1–78·4)	76·5 (75·1–78·0)	0·251
Intermediate risk (III)		5·3 (4·9–5·6)	5·9 (5·4–6·4)	4·8 (4·5–5·1)	< 0·001
High risk (IV + V + VI + VII)		17·9 (16·9–18·8)	16·9 (16·1–17·7)	18·7 (17·4–20·0)	0.001

^a^Weighted estimates ^b^After estimation of pre-treatment BP for those on BP lowering treatment

*CVD* cardiovascular disease, *SBP* systolic blood pressure, *DBP* diastolic blood pressure, *BP* blood pressure

### Classification of CVD risk

**Fig. 1:**
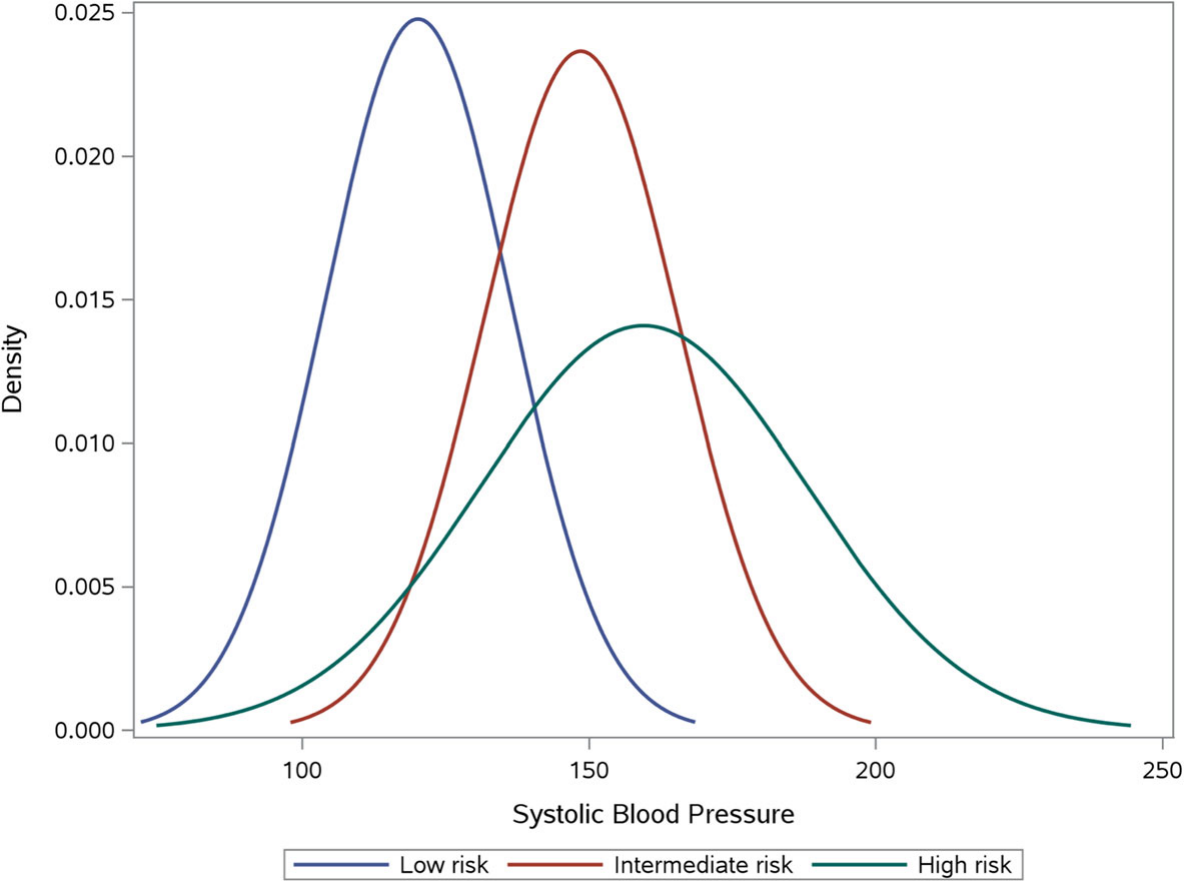
Frequency density of systolic blood pressure stratified by estimated 10-year CVD risk

Four percent of the study population reported established CVD. After imputing pre-treatment BP levels in patients already on treatment, 11·8% had “hypertension” using a BP threshold of 160/100 mmHg and 29.9% had “hypertension” using a BP threshold of 140/90 mmHg. The proportions having low, intermediate and high 10-year CVD risk were 76·8%, 5·3%, and 17·9%, respectively. Fewer individuals than may be expected were classified at intermediate risk; this was largely a result of those with BP > 160/100 mmHg being automatically classified as high risk. There was substantial overlap in systolic BP across CVD risk categories (Fig. 1).

### Blood pressure management

**Fig. 2:**
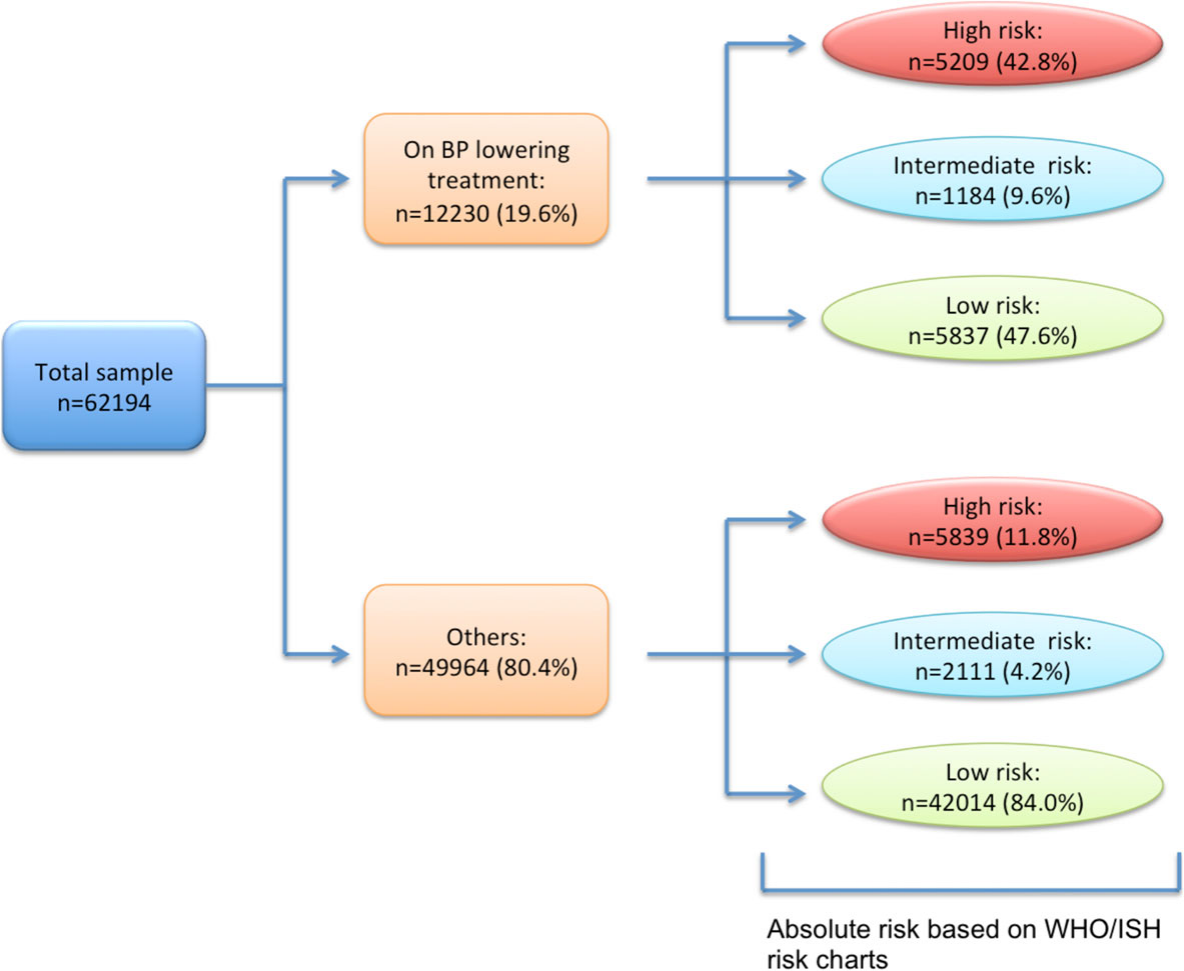
Estimated 10-year CVD risk distribution

A total of 12,230 individuals (19·6%) were taking BP lowering medication at the time of data collection. Imputing pre-treatment BP levels, 47·6% of those currently treated were classified as low risk (Fig. 2). Conversely, 4·2% and 11·8% of untreated individuals were estimated to be at intermediate or high risk, respectively.

### Comparison of treatment paradigms

If the population were completely untreated, an estimated 9442 CVD events would occur over ten years. Currently, approximately one in five individuals receive BP lowering treatment.

#### Comparison of alternate strategies to current practice

Targeting all intermediate- or high-risk individuals would result in a slightly greater proportion being treated, but would almost double the number of events averted over ten years (5·3% vs. 9·9%; Table 2). If only high-risk individuals were targeted for treatment, there would be a 9% reduction in total population treated, but would lead to 62·7% greater number of averted events. Implementation of NPCDCS guidelines would result in the treatment of a similar number of individuals, but with a 77·3% increase in the number of events averted.

**Table 2:** – Comparison of treatment paradigms for BP lowering^a^

Treatment paradigm for BP lowering	Population treated % (95% CI)	CVD events averted over 10 years due to treatment n (95% CI)	Estimated percentage reduction of CVD events averted over 10 years compared to no treatment† % (95% CI)	Percentage change in number treated compared to current practice % (95% CI)	Percentage change in number of events averted over 10 years compared to current practice % (95% CI)	Percentage change in number treated compared to “hypertension” paradigm % (95% CI)	Percentage change in number of events averted over 10 years compared to “hypertension” paradigm % (95% CI)
Current practice	19·6 (18·4–20·9)	502 (471–533)	5·3 (5·1–5·5)	–	–	–	–
Treatment of “hypertension” (BP > 140/90 mmHg)	32·6 (30·8–34·5)	869 (820–919)	9·2 (9·0–9·5)	+ 66·2 (65·8–66·5)	+ 74·2 (72·5–76·0)	–	–
Treatment according to NPCDCS guidelines	21·0 (19·9–22·2)	886 (838–934)	9·4 (9·1–9·7)	+ 7·2 (6·9–7·4)	+ 77·3 (75·6–79·0)	−35.5 (−35.2--35.8)	+ 1.7 (1.4–2.2)
Treatment of all at intermediate and high risk	23·2 (22·0–24·3)	936 (889–983)	9·9 (9·7–10·2)	+ 17·9 (17·6–18·2)	+ 87·2 (85·8–88·5)	−29.1 (−28.8--29.3)	+ 7.4 (6.7–8·3)
Treatment of all at high risk	17·9 (16·9–18·8)	812 (768–856)	8·6 (8·4–8·9)	−9·0 (−8·8--9·2)	+ 62·7 (60·7–64·6)	−45.2 (−44.9--45.5)	−6.6 (−5.9--7.4)
Treatment of all above 45 years of age	76·5 (75·4–77·6)	1233 (1216–1250)	13·0 (12·7–13·3)	+ 289·5 (289.2–289.8)	+ 145·7 (144.6–146.8)	+ 134.4 (133.7–135.6)	+ 41.0 (37.1–45.9)
Treatment of all above 55 years of age	44·5 (43·2, 45·8)	909 (883–935)	9·6 (9·3–9·9)	+ 126·6 (126.3–126.9)	+ 80·7 (78.9–82.4)	+ 36.4 (36.1–36.7)	+ 3.7 (3.2–4.3)

^a^Weighted estimates †Estimated number of CVD events over 10 years in the untreated population is 9442. This estimate is based on the population risk distribution after adjustment of BP levels in those on BP lowering treatment

*BP* blood pressure, *CVD* cardiovascular disease, *NPCDCS* National Program on prevention and control of Cancer, Diabetes, Cardiovascular diseases and Stroke

#### Comparison of alternate strategies to ‘hypertension’ strategy

Treatment of all intermediate- and high-risk individuals would result in 29.1% reduction in proportion of population treated with 7.4% greater number of averted events. Treatment as per the NPCDCS guidelines would lead to a similar number of events averted but would require 35.5% lesser number of individuals treated. If only high risk individuals were treated, there would be a 45.2% reduction in total population treated and 6.6% reduction in the number of events averted.

The cost effectiveness estimates found that treatment of all at high risk relative to current practice was cost saving (dominant). Treatment as per the NPCDCS guidelines (ICER = 27/DALY averted) and treatment of all at intermediate and high risk (ICER = 73.2/DALY averted) were each found to be highly cost effective (Table 3). These conclusions were based on the World Health Organization benchmarks (*highly cost effective*: ICER < one time per capital gross national income ($1570); *cost-effective*: ICER < three times per capital gross national income ($4710)) [31, 32].

**Table 3:** – Cost effectiveness of different treatment paradigms for BP lowering relative to current practice

Treatment paradigm for BP lowering	Population treated n (%)	CVD events averted over 10 years due to treatment	Treatment costs for patients at high risk of CVD (USD millions)	Hospitalization costs for CVD events (USD millions)	Incremental cost relative to current practice (USD millions)	Incremental CVD events averted relative to current practice	Incremental cost per CVD event averted relative to current practice (USD)	Incremental cost per DALY averted relative to currentpractice (1 CVD event = 22.5 DALYs)^a^
Current practice	12,190 (19.6)	502	4.4	0.10				
Treatment of “hypertension” (BP > 140/90 mmHg)	13,061 (32.6)	869	7.3	0.18	2.8	367	7723.0	342.9
Treatment according to NPCDCS guidelines	13,061 (21.0)	886	4.7	0.18	0.2	384	608.3	27.0
Treatment of all at intermediate and high risk	14,429 (23.2)	936	5.2	0.19	0.7	434	1649.2	73.2
Treatment of all at high risk	11,133 (17.9)	812	4.0	0.17	−0.4	310	Cost saving	Cost saving
Treatment of all above 45 years of age	47,578 (76.5)	1233	17.1	0.26	12.6	731	17,219.9	764.7
Treatment of all above 55 years of age	27,676 (44.5)	909	9.9	0.19	5.5	407	13,434.9	596.6

^a^The average burden associated with each cardiovascular event was calculated by dividing total burden in terms of disability adjusted life year (DALYs) lost due to coronary heart diseases with the total number of acute coronary events per year in India (28.6/1.27) [28, 29]

*BP* blood pressure, *CVD* cardiovascular disease, *NPCDCS* National Program on prevention and control of Cancer, Diabetes, Cardiovascular diseases and Stroke, *USD* The United States dollar, *DALY* Disability-adjusted life year

### Sensitivity analyses

Varying the major assumptions used in these analyses resulted in minimal to modest changes to estimates of the treated population size and numbers of events averted, however the differences between treatment paradigms remained broadly similar (Tables 1–6, online Appendix).

## Discussion

There are three important findings from this large cross-sectional study. First, a substantial proportion of this rural Indian population is at elevated risk of CVD. Second, many low-risk individuals currently receive BP lowering therapy, while treatment gaps among intermediate- and high-risk populations remain. Third, the widespread implementation of Indian guidelines that encourage an absolute risk approach to BP lowering treatment is highly cost-effective and in the specific case of treating all at high risk, cost-saving. Through this approach many more CVD events could be averted by treating fewer individuals, as well as significant amounts of money saved, if current practice was replaced with a strategy that only treats individuals at high CVD risk, regardless of BP level.

The finding of substantial CVD risk-factor burden in a rural Indian population is no longer surprising and reflects a rapid epidemiological transition that is affecting disadvantaged groups in low- and middle-income countries globally [7, 19]. The threat that CVD and other non-communicable diseases pose is now widely recognized with the World Health Assembly’s adoption of Global Action Plan for Prevention and Control of Non-communicable Diseases 2013–2020 [33]. This includes a global “25 by 25” goal – achieving 25% relative reduction in overall mortality from CVD, cancer, diabetes, and chronic respiratory diseases by 2025. Four of the nine targets within the Global Action Plan directly relate to control and management of BP related disease – namely; reducing population intake of salt/sodium; reducing or containing BP; essential drug availability; and appropriate drug therapy to prevent heart attack and stroke. Cost-effective strategies to identify and provide appropriate BP lowering drug treatment to individuals are therefore crucial to achieving at least some of these targets.

The advantages of basing treatment decisions on an individual’s CVD risk, rather than using an often-arbitrarily defined threshold level of an individual risk-factor have been long recognized [13, 19, 25, 34]. A recent large meta-analysis of BP lowering trials further strengthens the rationale for taking a risk-based approach, demonstrating similar CVD event protection regardless of initial BP level [25]. Therefore, as expected, the greatest absolute benefits of treatment accrued to those individuals at highest baseline risk. Strategies for identifying and treating individuals at high CVD risk are particularly important in low- and middle-income countries with major resource constraints and large under-treated populations [35]. Despite guidelines globally having an increased focus on absolute CVD risk-based approaches to blood pressure control [25], most practice appears to be entrenched on traditional paradigms that solely treat “hypertension”.

Our data indicate that current treatment in rural Andhra Pradesh is primarily driven by BP level rather than risk level, with approximately one-half of those on treatment being low risk, and only about 45% of individuals at high risk taking BP lowering drugs. The NPCDCS program in India, introduced in 2010, has largely taken a risk-based approach; further implementation of these recommendations would avert more events with the same proportion of the population currently being treated. Approximately 1.7 million events in individuals 40 years and above in Andhra Pradesh would be averted when compared to 0.9 million events, if the NPCDCS program recommendations are followed. Our data indicate that other risk-based approaches may be even more cost-efficient, an important consideration if available drug resources are severely constrained. We show that a similar number of CVD events could be averted as with current treatment, but with only half of the number of individuals requiring treatment using a 10-year absolute CVD risk threshold of 30%, this means 14 patients would require drug treatment for ten years to avert a CV event compared to 26 with current practice. Similar conclusions have been reached in studies in high-income countries [36, 37], but to the best of our knowledge this is the first-time comprehensive population-based data in a low- and middle-income country have established the advantages of an absolute risk approach to BP management.

The strengths of this study include collection of data on risk factors for CVD in virtually all eligible individuals in 54 villages of a south Indian state, likely to be generalizable to much of rural India. Robust, reproducible methods of data collection were used to collect these data. However, there are also some limitations. While only 13% of those eligible did not participate, these individuals were on average younger and more likely to be male than study participants. The former might have resulted in an over-estimation of population levels of CVD risk, however the opposite is true of the latter. The effectiveness of an absolute-risk approach relies on accurate CVD risk-assessment tools. While it has been adopted by NPCDCS, the performance of the WHO-ISH charts has not been adequately evaluated in the Indian context, and no other validated tools currently exist. The use of random (rather than fasting) blood glucose measurements (also adopted by the regular screening processes in NPCDCS) may have resulted in under-diagnosis of diabetes and thus under-estimation of the size of the high-risk population.

### Study limitations

We have made several assumptions in describing the effects of different treatment paradigms, based on meta-analyses of relevant clinical trials [38]. However sensitivity analyses indicate our conclusions remain robust to varying assumptions (please see Additional file 1). It should also be noted that treatment of other CVD risk factors was not considered in these analyses. In relation to use of other medications, recent studies in similar communities have demonstrated that even in the context of secondary prevention, the use of statins (6% in coronary heart disease and 1% in stroke), and antiplatelet therapy (19·4% in coronary heart disease and 11·8% in stroke) is very low, improved coverage with these medicines could further reduce events [39]. In the absence of other locally developed algorithms, the WHO/ISH risk charts for SEAR-D region were used to define the CVD risk in this study. These risk charts are in the process of being replaced because of concerns about their calibration in the target population. Newer risk charts will not likely influence the reported ranking of the effectiveness of different interventions, but could influence the predicted absolute numbers of events prevented and the cost-effectiveness estimates. In generating cost effectiveness estimates we applied data on India-specific average costs of pills and DALYs from the published literature to provide an indication of the investment case for each of the strategies. Although the estimates are crude, any error was unlikely to have affected the reported ranking of different interventions and findings that indicated overwhelmingly the economic arguments for shifts in treatment paradigm away from current practice. Finally, the study did not recruit individuals above 85 years, which is likely to have excluded few individuals, but a greater proportion at higher risk.

## Conclusion

An absolute risk based approach is important for preventing BP related diseases, particularly in low- and middle-income countries where resource constraints are a major barrier to CVD prevention. Guidelines are changing, but it will take concerted effort to change the decades-old paradigm of single-risk factor screening and management. In this study, we present an emphatic case for doing so based on population health outcomes and economic criteria. For the risk-based approach to be maximally effective, development and use of tools that easily and accurately predict an individual’s absolute risk of CVD is an urgent priority. Even if such tools were available, it is likely that healthcare providers will be slow to adopt new paradigms of treatment in the presence of numerous health system barriers, but unless funders and policy makers very rapidly re-orient “hypertension” programmes to CVD risk management, the likelihood of achieving some of the 25 × 25 targets will be significantly compromised.

## Additional file

Additional file 1: **Tables S1-S6.** We have made several assumptions in describing the effects of different treatment paradigms, based on meta-analyses of relevant clinical trials. The additional file 1 consists results of sensitivity analyses conducted to indicate our conclusions remain robust to varying assumptions. (DOCX 34 kb)

## Abbreviations

BP: Blood Pressure
CVD: Cardiovascular disease risk
DALYS: disability adjusted life years
NPCDCS: National Program on prevention and control of Cancer, Diabetes, Cardiovascular diseases, and Stroke
PHCs: Primary Healthcare Centers
SEAR D: South-East Asian Region-D
WHO/ISH: World Health Organization/ International Society for Hypertension
