# Supplementary material for: Cardiovascular disease risk and comparison of different strategies for blood pressure management in rural India
Source: BMC Public Health. 2018 Nov 15;18:1264. doi: 10.1186/s12889-018-6142-x (PMC6238360; doi:10.1186/s12889-018-6142-x)
Supplement: Supplementary file 1 — Tables S1-S6. We have made several assumptions in describing the effects of different treatment paradigms, based on meta-analyses of relevant clinical trials. The additional file 1 consists results of sensitivity analyses conducted to indicate our conclusions remain robust to varying assumptions. (DOCX 34 kb) [file 12889_2018_6142_MOESM1_ESM.docx]

**Supplementary File**

**Table S1-** Comparison of treatment paradigms for BP lowering, assuming average risk for the high risk population is 40%, maintaining all other assumptions as in Table 2*

| **Treatment paradigm for BP lowering** | **% Population treated**  **% (95% CI)** | **CVD events averted over 10 years due to treatment**  **n (95% CI)** | **Estimated percentage reduction of CVD events averted over 10 years due to treatment compared to no treatment†**  **% (95% CI)** | **Percentage change in number treated compared to current practice**  **% (95% CI)** | **Percentage change in number of events averted over 10 years compared to current practice**  **% (95% CI)** |
| --- | --- | --- | --- | --- | --- |
| Current practice | 19·6  (18.4-20·9) | 428  (401-454) | 5.1  (4.9-5.3) | - | - |
| Treatment of “hypertension” (BP >140/90 mmHg) | 32.6  (30.8-34.5) | 735  (693-777) | 8.8  (8.5-9.1) | +66.2  (65.8-66.5) | +72.8  (70.9-74.7) |
| Treatment according to NPCDCS guidelines | 21.0  (19·9-22.2) | 731  (691-770) | 8.7  (8.4-9.0) | +7·2  (6.9-7.4) | +71.5  (69.5-73.5) |
| Treatment of all at intermediate and high risk | 23.2  (22.0-24.3) | 781  (741-820) | 9.3  (9.0-9.6) | +17.9  (17.6-18.2) | +83.1  (81.5-84.8) |
| Treatment of all at high risk | 17.9  (16·9-18.8) | 657  (621-693) | 7.8  (7.6-8.1) | -9.0  ((-8.8)-(-9.2)) | +54.4  (52.2-56.6) |

*Weighted estimates

†Estimated number of CVD events over 10 years in the untreated population is 8412. This estimate is based on the population risk distribution after adjustment of BP levels in those on BP lowering treatment.

BP – blood pressure; CVD – cardiovascular disease; NPCDCS – National Program on prevention and control of Cancer, Diabetes, Cardiovascular diseases and Stroke

**Table S2-** Comparison of treatment paradigms for BP lowering, assuming average risk for the high risk population is 60%, maintaining all other assumptions as in Table 2*

| **Treatment paradigm for BP lowering** | **% Population treated**  **% (95% CI)** | **CVD events averted over 10 years due to treatment**  **n (95% CI)** | **Estimated percentage reduction of CVD events averted over 10 years compared to no treatment†**  **% (95% CI)** | **Percentage change in number treated compared to current practice**  **% (95% CI)** | **Percentage change in number of events averted over 10 years compared to current practice**  **% (95% CI)** |
| --- | --- | --- | --- | --- | --- |
| Current practice | 19·6  (18.4-20·9) | 575  (540-610) | 5.5  (5.3-5.7) | - | - |
| Treatment of “hypertension” (BP >140/90 mmHg) | 32.6  (30.8-34.5) | 1002  (945-1059) | 9.6  (9.4-9.9) | +66.2  (65.8-66.5) | +75.3  (73.7-76.9) |
| Treatment according to NPCDCS guidelines | 21.0  (19·9-22.2) | 1040  (983-1096) | 10.0  (9.7-10.2) | +7·2  (6.9-7.4) | +81.6  (80.1-83.0) |
| Treatment of all at intermediate and high risk | 23.2  (22.0-24.3) | 1090  (1035-1145) | 10.4  (10.2-10.7) | +17.9  (17.6-18.2) | +90.2  (89.1-91.3) |
| Treatment of all at high risk | 17.9  (16·9-18.8) | 967  (9914-1019) | 9.3  (9.0-9.5) | -9.0  ((-8.8)-(-9.2)) | +68.8  (67.1-70.6) |

*Weighted estimates

†Estimated number of CVD events over 10 years in the untreated population is 10473. This estimate is based on the population risk distribution after adjustment of BP levels in those on BP lowering treatment.

BP – blood pressure; CVD – cardiovascular disease; NPCDCS – National Program on prevention and control of Cancer, Diabetes, Cardiovascular diseases and Stroke

**Table S3-** Comparison of treatment paradigms for BP lowering, assuming relative risk reduction from BP lowering drug therapy of 10%, maintaining all other assumptions as in Table 2*

| **Treatment paradigm for BP lowering** | **% Population treated**  **% (95% CI)** | **CVD events averted over 10 years due to treatment**  **n (95% CI)** | **Estimated percentage reduction of CVD events averted over 10 years compared to no treatment†**  **% (95% CI)** | **Percentage change in number treated compared to current practice**  **% (95% CI)** | **Percentage change in number of events averted over 10 years compared to current practice**  **% (95% CI)** |
| --- | --- | --- | --- | --- | --- |
| Current practice | 19·6  (18.4-20·9) | 334  (313-355) | 3.5  (3.4-3.7) | - | - |
| Treatment of “hypertension” (BP >140/90 mmHg) | 32.6  (30.8-34.5) | 579  (546-612) | 6.2  (5.9-6.4) | +66.2  (65.8-66.5) | +74.2  (72.1-76.4) |
| Treatment according to NPCDCS guidelines | 21.0  (19·9-22.2) | 590  (558-622) | 6.3  (6.0-6.5) | +7.2  (6.9-7.4) | +77.3  (75.2-79.3) |
| Treatment of all at intermediate and high risk | 23.2  (22.0-24.3) | 624  (592-655) | 6.6  (6.4-6.9) | +17.9  (17.6-18.2) | +87.2  (85.5-88.8) |
| Treatment of all at high risk | 17.9  (16·9-18.8) | 541  (512-571) | 5.8  (5.5-6.0) | -9.0  ((-8.8)-(-9.2)) | +62.7  (60.3-65.1) |

*Weighted estimates

†Estimated number of CVD events over 10 years in the untreated population is 9442. This estimate is based on the population risk distribution after adjustment of BP levels in those on BP lowering treatment.

BP – blood pressure; CVD – cardiovascular disease; NPCDCS – National Program on prevention and control of Cancer, Diabetes, Cardiovascular diseases and Stroke

**Table S4-** Comparison of treatment paradigms for BP lowering, assuming relative risk reduction from BP lowering drug therapy of 20%, maintaining all other assumptions as in Table 2*

| **Treatment paradigm for BP lowering** | **% Population treated**  **% (95% CI)** | **CVD events averted over 10 years due to treatment**  **n (95% CI)** | **Estimated percentage reduction of CVD events averted over 10 years compared to no treatment†**  **% (95% CI)** | **Percentage change in number of treated compared to current practice**  **% (95% CI)** | **Percentage change in number of events averted over 10 years compared to current practice**  **% (95% CI)** |
| --- | --- | --- | --- | --- | --- |
| Current practice | 19·6  (18·4-20·9) | 669  (627-710) | 7.1  (6.8-7.3) | - | - |
| Treatment of “hypertension” (BP >140/90 mmHg) | 32.6  (30.8-34.5) | 1158  (1092-1224) | 12.3  (12.0-12.6) | +66.2  (65.8-66.5) | +74.2  (72.7-75.8) |
| Treatment according to NPCDCS guidelines | 21.0  (19·9-22.2) | 1180  (1116-1244) | 12.5  (12.2-12.9) | +7.2  (6.9-7.4) | +77.3  (75.8-78.7) |
| Treatment of all at intermediate and high risk | 23.2  (22.0-24.3) | 1247  (1184-1311) | 13.2  (12.9-13.6) | +17.9  (17.6-18.2) | +87.2  (86.0-88.4) |
| Treatment of all at high risk | 17·9  (16·9-18.8) | 1083  (1023-1142) | 11.5  (11.2-11.8) | -9.0  ((-8.8)-(-9.2)) | +62.7  (61.0-64.4) |

*Weighted estimates

†Estimated number of CVD events over 10 years in the untreated population is 9442. This estimate is based on the population risk distribution after adjustment of BP levels in those on BP lowering treatment.

BP – blood pressure; CVD – cardiovascular disease; NPCDCS – National Program on prevention and control of Cancer, Diabetes, Cardiovascular diseases and Stroke

**Table S5-** Comparison of treatment paradigms for BP lowering, assuming pre-treatment levels were higher by 10 mmHg for SBP and 5 mmHg for DBP, among those already taking BP lowering drugs, maintaining all other assumptions as in Table 2*

| **Treatment paradigm for BP lowering** | **% Population treated**  **% (95% CI)** | **CVD events averted over 10 years due to treatment**  **n (95% CI)** | **Estimated percentage reduction of CVD events averted over 10 years compared to no treatment†**  **% (95% CI)** | **Percentage change in number population treated compared to current practice**  **% (95% CI)** | **Percentage change in number of events averted over 10 years compared to current practice**  **% (95% CI)** |
| --- | --- | --- | --- | --- | --- |
| Current practice | 19.6  (18.4-20.9) | 515  (483-547) | 5.4  (5.2-5.6) | - | - |
| Treatment of “hypertension” (BP >140/90 mmHg) | 33.5  (31.6-35.4) | 893  (842-943) | 9.4  (9.1-9.7) | +70.7  (70.3-71.2) | +74.3  (72.3-76.3) |
| Treatment according to NPCDCS guidelines | 21.5  (20.4-22.7) | 903  (854-951) | 9.5  (9.2-9.8) | +9.7  (9.4-10.0) | +76.0  (74.0-78.0) |
| Treatment of all at intermediate and high risk | 23.6  (22.4-24.8) | 953  (904-1001) | 10.0  (9.7-10.3) | +20.4  (20.0-20.8) | +85.6  (84.0-87.2) |
| Treatment of all at high risk | 18.1  (17.1-19.1) | 824  (779-869) | 8.7  (8.4-9.0) | -7.7  ((-7.4)-(-7.9)) | +60.7  (58.4-62.9) |

*Weighted estimates

†Estimated number of CVD events over 10 years in the untreated population is 9533. This estimate is based on the population risk distribution after adjustment of BP levels in those on BP lowering treatment.

BP – blood pressure; CVD – cardiovascular disease; NPCDCS – National Program on prevention and control of Cancer, Diabetes, Cardiovascular diseases and Stroke

**Table S6-** Comparison of treatment paradigms for BP lowering, assuming pre-treatment levels were higher by 5 mmHg for SBP and 3 mmHg for DBP, among those already taking BP lowering drugs, maintaining all other assumptions as in Table 2*

| **Treatment paradigm for BP lowering** | **% Population treated**  **% (95% CI)** | **CVD events averted over 10 years due to treatment**  **n (95% CI)** | **Estimated percentage reduction of CVD events averted over 10 years compared to no treatment†**  **% (95% CI)** | **Percentage change in number treated compared to current practice**  **% (95% CI)** | **Percentage change in number of events averted over 10 years compared to current practice**  **% (95% CI)** |
| --- | --- | --- | --- | --- | --- |
| Current practice | 19.6  (18.4-20.9) | 471  (442-500.1) | 5.1  (4.9-5.3) | - | - |
| Treatment of “hypertension” (BP >140/90 mmHg) | 31.9  (30.1-33.8) | 828  (780-876) | 9.0  (8.7-9.3) | +62.6  (62.2-63.0) | +76.9  (75.2-78.7) |
| Treatment according to NPCDCS guidelines | 20.3  (19.2-21.4) | 848  (802-894) | 9.2  (8.9-9.5) | +3.3  (3.1-3.4) | +76.0  (74.0-78.0) |
| Treatment of all at intermediate and high risk | 22.5  (21.3-23.6) | 900  (855-945) | 9.8  (9.5-10.0) | +14.4  (14.1-14.7) | +85.6  (84.0-87.2) |
| Treatment of all at high risk | 17.0  (16.1-18.0) | 773  (730-817) | 8.4  (8.1-8.7) | -13.2  ((-13.0)-(-13.5)) | +65.0  (63.0-67.0) |

*Weighted estimates

†Estimated number of CVD events over 10 years in the untreated population is 9533. This estimate is based on the population risk distribution after adjustment of BP levels in those on BP lowering treatment.

BP – blood pressure; CVD – cardiovascular disease; NPCDCS – National Program on prevention and control of Cancer, Diabetes, Cardiovascular diseases and Stroke
